# Novel Non-Coding Transcript in *NR4A3* Locus, LncNR4A3, Regulates RNA Processing Machinery Proteins and *NR4A3* Expression

**DOI:** 10.3389/fonc.2020.569668

**Published:** 2020-11-23

**Authors:** Ada Congrains, Fernanda Soares Niemann, Adriana Da Silva Santos Duarte, Karla Priscila Vieira Ferro, Sara Teresinha Olalla-Saad

**Affiliations:** Hematology and Transfusion Medicine Center, University of Campinas, Campinas, Brazil

**Keywords:** NR4A3, RNA processing, long non-coding RNA, myeloid malignancy, acute myeloid leukemia

## Abstract

*NR4A3* is a key tumor suppressor in myeloid malignancy, mice lacking both *NR4A1* and family member *NR4A3* rapidly develop lethal acute myeloid leukemia (AML). We identified a long non-coding transcript in the *NR4A3* locus and pursued the characterization of this anonymous transcript and the study of its role in leukemogenesis. We characterized this novel long non-coding transcript as a sense polyadenylated transcript. Bone marrow cells from AML patients expressed significantly reduced levels of lncNR4A3 compared to healthy controls (controls = 15, MDS= 20, p=0.05., AML= 21, p<0.01). Expression of *NR4A3*, as previously reported, was also significantly reduced in AML. Interestingly, the expression of both coding and non-coding transcripts was highly correlated (Pearson R = 0.3771, P<0.01). Transient over-expression of LncNR4A3 by nucleofection led to an increase in the RNA and protein level of *NR4A3*, reduction of proliferation in myeloid cell lines K-562 and KG1 (n=3 and 2 respectively, p<0.05) and reduced colony formation capacity in primary leukemic cells. A mass spectrometry-based quantitative proteomics approach was used to identify proteins dysregulated after lncNR4A3 over-expression in K-562. Enrichment analysis showed that the altered proteins are biologically connected (n=4, p<0.001) and functionally associated to RNA binding, transcription elongation, and splicing. Remarkably, we were able to validate the most significant results by WB. We showed that this novel transcript, lncNR4A3 regulates *NR4A3* and we hypothesize this regulatory mechanism is mediated by the modulation of the RNA processing machinery.

## Introduction

*NR4A3/NOR-1* is a member of the NR4A orphan nuclear receptor subfamily. This subfamily comprises three closely related members: *NR4A1* (also known as Nur77, TR3, or NGFI-B), *NR4A2* (also known as Nurr1, RNR-1, or TONOR), and *NR4A3* (also known as NOR-1 or MINOR). NR4As dysregulation has been associated with a wide range of conditions including atherosclerosis, diabetes, and several malignancies (1–7).

The role of *NR4A1* and *NR4A3* in myeloid malignancy is particularly relevant. *NR4A1/NR4A3* knock-out mice rapidly develop lethal acute myeloid leukemia (AML) (8, 9) and reduced dosage of these genes, in mice, leads to a phenotype that recapitulates myelodysplastic syndrome (MDS), a hematologic disorder with increased susceptibility to AML. Additionally, leukemic blasts from AML patients have reduced expression of *NR4A1/NR4A3* genes (9). This evidence strongly supports the hypothesis that the loss of tumor suppressors *NR4A1/3* is a key initiating step in leukemic transformation. Strategies aiming to block the inactivation of these transcription factors would hold great potential in the treatment of AML and MDS. However, the mechanisms that lead to their inactivation remain elusive.

Long non-coding RNAs are increasingly recognized as master regulators of cellular function in health and disease (10–14). These non-coding transcripts are involved in virtually all steps of genetic regulation. They recruit chromatin modifying proteins (15) and transcription factors (16), hijack the splicing (17) and translation machinery (11), sequester miRNAs (18), among other functions. Previous work of our group identified a long non-coding RNA in the *NR4A3* locus expressed in hematopoietic stem cells from myelodysplastic syndrome patients (19). Due to the important role of *NR4A3* gene in myeloid malignancy, we pursued the functional characterization of this transcript.

A long non-coding RNA encoded in the *NR4A3* locus is an interesting candidate to explore cis regulation upon *NR4A3*. Here, we characterized this hitherto unknown transcript, evaluated its expression in patient samples, functionally studied its role in NR4A3 locus regulation and used a mass-spectrometry-based proteomics approach to identify the targets of lncNR4A3.

## Patients, Materials, and Methods

### Patients

Samples from patients with previously untreated AML and MDS by World Health Organization (WHO) criteria, were used in this study. Diagnosis was confirmed by cytologic examination of blood and bone marrow (patient characteristics shown in **Table 1**). Mononuclear cells were isolated by Ficoll-Hypaque separation of total bone marrow (BM). Samples from MDS patients (12 males, 8 females, median age: 74, range: 31–86 years) and AML patients (12 males, 9 females, median age, 59 years, range, 22–88 years) were collected at the time of diagnosis and BM mononuclear cells of 15 controls (12 males, 3 females, median age, 30 years, range, 15–47 years) were obtained from bone marrow donors. French-American-British (FAB) classification of the patients is presented in **Table 1**. All patients were diagnosed between 2009 and 2014 at the hematology and transfusion medicine center, University of Campinas. Bone marrow mononuclear cells for the nucleofection experiments were obtained from BM aspirates of two AML patients. The mononuclear cell fraction was separated as described above. One patient had more than 80% CD34+ cells and cells were directly used for the experiments and for the other patient, CD34+ cells were separated using Indirect CD34 MicroBead Kit, Miltenyi Biotech GmbH, Germany. All participants gave written informed consent to the study; procedures were approved by the University Ethics Committee (number CEP1209/2011) and all methods were in accordance with the relevant guidelines and regulations.

**Table 1:** Clinical characteristics of patients and healthy controls.

Characteristics patients and healthy controls		
	Number	
**Controls**	15	
Sex (male/female)	12/3	
Age median [range]	30 [15–47]	
**MDS cases**	20	
Sex (male/female)	12/8	
Age median [range]	59 [31–86]	
% of blasts in BM, mean	5.9	
*FAB classification*		
Low risk (RA/RARS)	4/4	
High risk (RAEB/RAEB-t)	11/1	
**AML cases**	21	
Sex (male/female)	12/9	
Age median [range]	59 [22–88]	
% of blasts in BM, mean	77.6	
FAB classification		
M0	1	
M1	6	
M2	5	
M3	2	
M4	4	
M5	2	
AML-MRC (secondary to MDS)	1	
**AML cases for nucleofection**	2	
	FAB classification	% of blasts in BM
Case 1	M3	95
Case 2	M4	59.6

RA, refractory anemia; RARS, refractory anemia with ringed sideroblasts; RAEB, refractory anemia with excess blasts; RAEB-t, refractory anemia with excess blasts in transformation; M0, undifferentiated acute myeloblastic leukemia; M1, acute myeloblastic leukemia with minimal maturation; M2, acute myeloblastic leukemia with maturation; M3, acute promyelocytic leukemia; M4, acute myelomonocytic leukemia; M5, acute monocytic leukemia; AML-MRC, acute myeloid leukemia with myelodysplasia-related changes.

### Nucleofection

After full characterization of lncNR4A3, we successfully cloned the transcript in pcDNA™3.1 (+) (Invitrogen) expression vector. The lncNR4A3 and empty vector were delivered into k-562, KG1 cells, and CD34+ hematopoietic cells by nucleofection using AMAXA nucleofector device (Lonza, Switzerland) and SF Cell Line Kit (for K-562 and KG1) and P3 Primary Cell Kit (for CD34+cells), (Lonza, Switzerland). 10^6^ cells were nucleofected with 2 µg of vector for K-562 and CD34+ cells and 4 µg for KG1 cells according to the optimized protocol provided by the manufacturer. After nucleofection cells CD34+ cells were resuspended in expansion medium StemSpan™, Stemcell Technologies (supplemented with 10ng/ml of IL3, IL6, FLT3, TPO), or methylcellulose medium (MethoCult™ Stemcell). Nucleofection efficiency was evaluated using 2µg of pmax-GFP and results are shown in **Supplementary Results**.

### Quantitative RT-PCR (qRT-PCR)

RNA was isolated from k-562, KG1, CD34+ cells and total bone marrow samples using Illustra RNAspin Mini Kit (GE Healthcare Life Sciences) following the manufacturer’s instructions. RNA quantification was performed in a NanoDrop spectrophotometer (ND-1000 Spectrophotometer). A total of 1 µg of RNA from each sample (except for CD34+ cells, from which samples less than 1µg RNA were obtained) was reverse transcribed into complementary DNA (cDNA) (RevertAid First Strand cDNA Synthesis Kit, Thermo Scientific) using random primers. For strand-specific PCR we used sequence specific primers for reverse transcription (complementary to positive and negative strands respectively), instead of dT oligos or random primers. These primers were designed near the expected ends of lncNR4A3.

Real-time PCR amplifications were performed on the ABI 7500 Sequence Detector System (Applied Biosystems) using SybrGreen PCR Master Mix (Applied Biosystems). Primers sequences are provided in **Supplementary Methods**.

### Viability Assay—CCK-8

For proliferation analysis, cell counting kit, CCK-8 (Dojindo Molecular Technologies, Inc.) assay was used. This viability assay uses a water-soluble tetrazolium salt, 2-(2-methoxy-4-nitrophenyl)-3-(4-nitrophenyl)-5-(2,4-disulfophenyl)-2H tetrazolium, monosodium salt (WST-8), which has been shown to have greater sensitivity than traditional assays such as MTT (20). Cells were seeded in 96-well plates at a density of 2x10^5^ cells/ml after nucleofection and were incubated at 37°C for further 48 and 72 h for K-562 and KG1 respectively. After that time, CCK-8 reagent was added to the well, following manufacturer’s instruction and cell viability was assessed by measurement of absorbance at 450nm, expressed relative to control empty-vector-nucleofected cells.

### Proteomic Analysis

Protein was extracted as described above and concentrations were determined by Bradford protein quantification assay. A total of 50 μg of protein were run in a sodium dodecyl sulfate-polyacrylamide gel electrophoresis (SDS-PAGE) and undergo reduction, alkylation, and in-gel digestion with trypsin (details in **Supplementary Methods**). Peptides were separated by C18 (100 mm 6,100 mm) RP-nanoUPLC (nanoACQUITY, Waters) coupled with a Q-Tof Premier Mass Spectrometer (Waters) with nanoelectrospray source at a flow rate of 0.6 ml/min.

For protein quantification, data was analyzed by Scaffold Q+ (version 4.4.3; Proteome Software, Inc., Portland, OR, USA) and set to a false discovery under 1%. Gene ontology enrichment was carried out using String software V10.5. Details of analysis in **Supplementary Methods**.

### Western Blotting

Cells were lysed in a buffer containing 100 mM Tris (pH 7.6), 1% Triton X-100, 150 mM NaCl, 0.1 mg aprotinin, 35 mg/ml PMSF 10 mM Na3VO4, 100 mM NaF, 10 mM Na4P2O7, and 4 mM EDTA (approximately 5 × 10^6^ cells). Samples were centrifuged at 4°C for 20 min to remove cell debris. Protein concentration was measured using the Bradford Assay (Bio-Rad). Laemmli buffer containing 100 mmol/L of dithiothreitol was added to the protein extracts and heated at 100°C for 5 min. Samples were run on a 10% SDS-PAGE. After the run, the proteins were transferred to nitrocellulose membranes (Millipore). Membranes were immunoblotted with NR4A3 (Abcam, ab41918), HnRNPK (Abcam, ab32969), SF3B2 (ProteinTech, 10919-1-AP), HnRNPA1 (ProteinTech, 11176-1-AP), PARP1 (Santa Cruz, sc-56197), Lamin B1 (Santa Cruz, sc-6127), and GAPDH (Santa Cruz, sc-32233) antibodies. K-562 protein samples were obtained from three independent experiments, all bands are shown in the **Supplementary Material**; however, only one patient sample of HSC CD34+ cells (which is a rare population of hematopoietic progenitors) was available for protein extraction. Band intensity was quantified using UVITEC alliance software.

### Statistical Analysis

Statistical analysis of the data was performed using R version 3.5.1 and GraphPad prism software. The patient’s data was analyzed by Student’s t-test (two-way) and statistical significance between two groups (controls *vs*. MDS, and controls *vs*. AML) is shown in the graphs. To measure correlation between lncNR4A3 and NR4A3, the Pearson coefficient was calculated, Pearson r and P-value are shown in the corresponding figure. For the functional experiments the significance of differences between two groups (empty vector and lncNR4A3) was estimated with Student’s t test and differences were considered statistically significant at the level of P < 0.05. Figures were plotted using “R” programing language (“ggplot2,” “cowplot,” “gridextra” packages). For screening of differently expressed proteins from the proteomic analysis, we used an in-house program developed in “R” 3.5.1 to apply ANOVA built-in function to all proteins detected; however, proteins with more than three missing spectrometry readings were excluded from the analysis.

## Results

### lncNR4A3 Characterization

**Figure 1:**
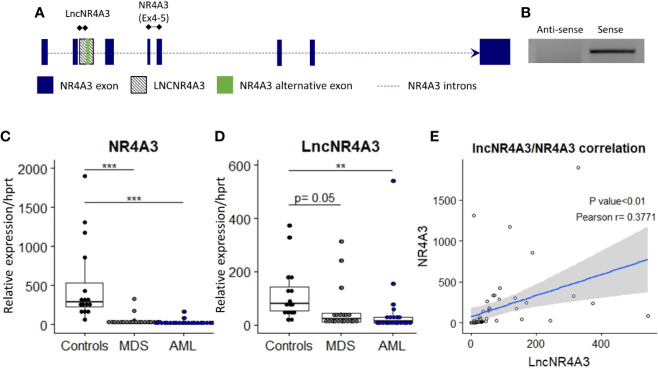
**(A)** Transcriptional map of the NR4A3 locus showing position of LncNR4A3 and primers used in this work. **(B)** EtdBr-staining bands in agarose gel electrophoresis from strand-specific PCR to identify the orientation of the transcript, lncNR4A3 is transcribed in the same orientation than NR4A3. **(C, D)** Quantitative RT-PCR (QRT-PCR) quantification of NR4A3 and long non-coding transcript, LncNR4A3, in normal bone marrow cells (NBM) of 15 controls, 20 myelodysplastic syndrome patients (MDS), and 21 acute myeloid leukemia patients bone marrows (AML). **(E)** Linear regression of the expression of LncNR4A3 plotted against NR4A3 expression and correlation analysis results (Pearson correlation P value, R coefficient). ** : <0.01, *** : <0.001.

To characterize lncNR4A3, we based on a partial sequence identified by Nakaya *et al*. and deposited in a dataset for partially intronic non-coding RNAs (21). Additionally, we performed a search for ESTs (expressed sequence tags) in the UCSC database in this region, identifying several human ESTs (Accession: BG539866, BG546553, BG570616, BF105874, BE502919, BE219816, AW204232, see **Supplementary Figure 1**). Since most PCR approaches are not direction-sensitive, we used strand-specific PCR to identify the orientation of the transcript, which is based on synthesis of cDNA using specific primers complementary to positive and negative strands of DNA near the expected ends of the transcript from both strands. This assay revealed that lncNR4A3 is transcribed in the same orientation than NR4A3, hence considered a sense transcript (**Figure 1B**). To characterize the complete sequence of lncNR4A3, we used primers near the putative 5′ and 3′ ends to perform rapid amplification of cDNA ends. Due to the difficulty to amplify the 3´end of this transcript, we suspected it did not have a poly A tail. We used oligo dT beads to enrich RNAs with poly A tails and collected RNA not bound to the beads as RNA depleted of polyadenylated transcripts. Unexpectedly, LncNR4A3 only amplified in the poly A enriched fraction for cDNA from K-562, suggesting LncNR4A3 is polyadenylated (see **Supplementary Figure 2**). After several attempts and methods (see **Supplementary Methods**) LncNR4A3 full sequence (deposited under NCBI GeneBank accession number MK510719) was identified by RACE. This sequence also matches the alignment of ESTs found in the region, supporting MK510719 is the full sequence of the transcript (see **Supplementary Figure 1**). LncNR4A3 was characterized as a polyadenylated, 1,214 bp transcript overlapping the second intron, and an alternative exon of the NR4A3 gene, see **Figure 1A**.

### Expression of lncNR4A3 and *NR4A3* Is Suppressed in Acute Myeloid Leukemia

The novel non-coding transcript, lncNR4A3 was significantly reduced in cells from the bone marrow of AML patients (controls= 15, MDS=20, AML=21), see **Figure 1D**. As previously reported (9), *NR4A3* is also reduced in bone marrow cells from MDS and AML patients (**Figure 1C**). Expression of lncNR4A3 and *NR4A3* were positively correlated (Pearson R = 0.3771, P<0.01, **Figure 1E**), supporting involvement of one in the expression of the other.

We also characterized the expression of lncNR4A3 in several myeloid and lymphoid cell lines, CD34+ hematopoietic stem cells, and non-hematopoietic cells lines HS5 and Hela (see **Supplementary Figure 4**). LncNR4A3 was detected in all cell types evaluated, but expression in myeloid malignant cell lines was extremely low compared to CD34+ HSCs (a normal hematopoietic progenitor cell population from umbilical cord blood) and HS5 (stromal cell line).

### LncNR4A3 Over-Expression Modulates the Expression of RNA-Binding Proteins and Particularly Members of the hnRNP Family

**Figure 2:**
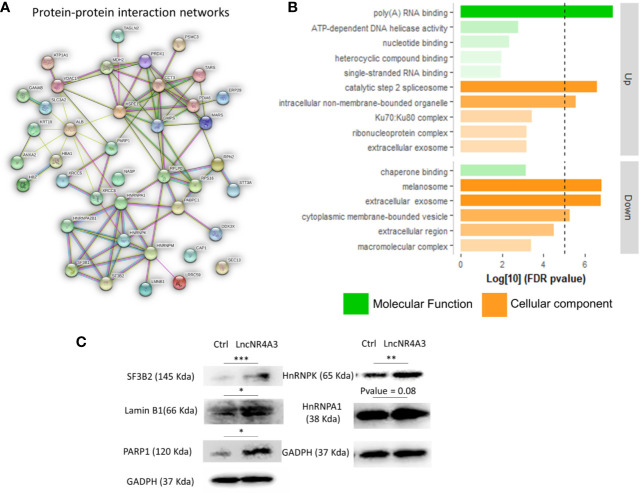
**(A)** Protein–protein interaction network, a weighted linkage graph constructed using STRING software, inputting the differentially expressed proteins identified in the proteomics analysis. **(B)** Histogram plotting the Log[10] of the p-values obtained from the gene ontology enrichment analysis, cellular component, and molecular function ontology Log[10]p-values were plotted for up-regulated and down-regulated proteins obtained from proteomics analysis, threshold of 5 is denoted by the dashed line. **(C)** Immunoblots confirming regulation of several target proteins identified by the MS-proteomics analysis in K-562 controls and lncNR4A3 over-expressing cells, blots from three independent experiments shown in **Supplementary Figure 10**, paired t-test was applied to stablish significant differences between optical density of the blots (*: <0.05, **: <0.01, ***<0.001).

We investigated the global effects of lncNR4A3 over-expression among the entire proteome of lncNR4A3 over-expressing K-562 cells compared to controls. Whole-cell lysates were processed and analyzed by Q-tof mass spectrometry as described in methods. A total of over 400 proteins were identified (see complete list in **Supplementary Data File**). Enrichment analysis using STRING software showed that the altered proteins are biologically connected (n=4, p<0.001, see protein-protein interaction network, **Figure 2A**) and functionally associated to RNA binding and processing and cellular components including the spliceosome (**Figure 2B**).

Pathways analysis rendered similar results, KEGG pointed to protein processing in endoplastic reticulum and spliceosome as second most significantly associated pathway, “Reactome” gave metabolism of RNA as most associated metabolic pathway and “Local String network cluster” pointed to messenger RNA (mRNA) splicing as associated pathway. The association of these pathways support the role of lncNR4A3 in RNA processing.

From this analysis 22 proteins were significantly up-regulated and 19 down-regulated (p<0.05). Among the regulated proteins there are four members of the hnRNP (heterogeneous nuclear ribonucleoproteins) family and three of them up-regulated (**Table 2**, **Figure 2C**). The most significantly enriched protein in the lncNR4A3-K-562 cells was the heterogeneous nuclear ribonucleoprotein K (p-value = 0.00058). We validated the upregulation of *hnRNPK* in K-562 cells and AML patient cells; however, no effect was detected in KG1 cell line (see **Supplementary Figure 9**). Heterogeneous nuclear ribonucleoproteins (hnRNPs) are involved in RNA translocation and processing, also considered splicing switches (22, 23). Poly (ADP) ribose polymerase (*PARP1*) upregulation was also validated, while this protein is most known for its role in DNA damage repair, it is also involved in alternative splicing and RNA elongation (24, 25). Among up-regulated proteins, western blotting confirmed increased expression of splicing factor 3 B2 (*SF3B2*), a component of the spliceosome machinery that promotes splicing. Remarkably, splicing defects are key in leukemogenesis and mutations in the genes encoding the splicing machinery proteins are common in hematologic malignancy (26, 27). Lamin B1 was also among the proteins up-regulated by LncNR4A3 over-expression, this protein is an important structural component of the nucleus and evidence shows it is crucial for RNA synthesis and proliferation (28, 29). And more interestingly, expression of NR4A3 is known to be regulated by RNA splicing and elongation (30).

**Table 2:** MS-proteomics analysis results.

Up-regulated				
Protein name	Control (mean)	LncNR4A3 (mean)	P-value (ANOVA)	Gene symbol
Heterogeneous nuclear ribonucleoprotein K	8	14.25	0.00058	HNRNPK
Isoform of O00571, ATP-dependent RNA helicase DDX3X	4.5	10.75	0.003531	DDX3X
Poly[ADP-ribose] polymerase 1	1	10.5	0.005331	PARP1
Isoform of P12956, X-ray repair cross-complementing protein 6	6	20.75	0.005752	XRCC6
GMP synthase [glutamine-hydrolyzing]	3.75	10	0.006819	GMPS
Isoform of P52272, heterogeneous nuclear ribonucleoprotein M	2.75	8.5	0.010682	HNRNPM
Isoform of P09651, heterogeneous nuclear ribonucleoprotein A1	2.5	6	0.011724	HNRNPA1
Isoform of Q06830, peroxiredoxin-1 (fragment)	10.33	14.33	0.013235	PRDX1
Leucine-rich repeat-containing protein 59	5.25	6.67	0.017570	LRRC59
Nuclear autoantigenic sperm protein	3	7.25	0.017570	NASP
Lamin-B1	2	5.5	0.020311	LMNB1
Splicing factor 3B subunit 1	0.75	3	0.0388027	SF3B1
X-ray repair cross-complementing protein 5	3.75	9	0.040405	XRCC5
Hemoglobin subunit zeta	4	8.33	0.040642	HBZ
Isoform of P49368, T-complex protein 1 subunit gamma	9.75	14.75	0.042462	CCT3
Isoform of P11940, polyadenylate-binding protein	2.75	7.75	0.0424626	PABPC1
Threonine–tRNA ligase, cytoplasmic	5.75	9.75	0.043422	TARS
Isoform of P17980, 26S proteasome regulatory subunit 6A (fragment)	1.5	3.67	0.044629	PSMC3
Isoform of Q13435, splicing factor 3B subunit 2	0.25	2.25	0.0446904	SF3B2
Transgelin-2 OS=homo sapiens	22	29.67	0.0452401	TAGLN2
Keratin, type I cytoskeletal 19	1	3.67	0.045318	KRT19
Adenylyl cyclase-associated protein 1	7.5	8.5	0.049825	CAP1
**Downregulated**				
Exosome RNA helicase MTR4	1	0	1,24E−29	MTREX
Isoform of P55735, protein SEC13 homolog	4	0.33	0.001222	SEC13
60S acidic ribosomal protein P0	13.75	9.67	0.003655	RPLP0
Methionine–tRNA ligase, cytoplasmic	9	4.75	0.00414	MARS
Protein disulfide-isomerase A6	27.75	12.5	0.004389	PDIA6
Isoform of Q14697, neutral alpha-glucosidase AB	21.25	5.25	0.0053375	GANAB
Annexin A2 OS=homo sapiens	10	2.67	0.006343	ANXA2
Sodium/potassium-transporting ATPase subunit alpha-1	11	1.75	0.010610	ATP1A1
Isoform of P62249, 40S ribosomal protein S16	5.33	4	0.016130	RPS16
Malate dehydrogenase, mitochondrial	54	34.33	0.018010	MDH2
Isoform of P02768, serum albumin	11.25	1.75	0.018507	ALB
Endoplasmic reticulum resident protein 29	14	6	0.020775	ERP29
Isoform of P08195, 4F2 cell-surface antigen heavy chain	6	1	0.0221184	SLC3A2
Hemoglobin subunit alpha	6	2.5	0.027172	HBA1
Heterogeneous nuclear ribonucleoproteins A2/B1	6.25	4	0.02933	HNRNPA2B1
Dolichyl-diphosphooligosaccharide–protein glycosyltransferase subunit 2	15.75	4.5	0.029406	RPN2
Dolichyl-diphosphooligosaccharide–protein glycosyltransferase subunit STT3A	3.75	1.25	0.030766	STT3A
Voltage-dependent anion-selective channel protein 1	12.75	2.75	0.0336062	VDAC1
10 kDa heat shock protein, mitochondrial	7.67	2.33	0.0474206	HSPE1

### Artificial Re-Expression of lncNR4A3 Leads to Increased Expression of NR4A3 Messenger RNA and NR4A3 Protein Level and Reduction of Proliferation in Myeloid Cell Lines K-562, KG1, and Primary Leukemic Cells

**Figure 3:**
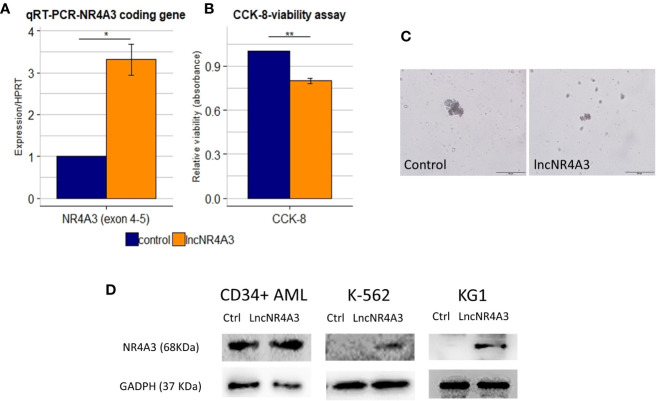
**(A)** Relative quantification by quantitative RT-PCR (qRT-PCR) of NR4A3 expression in LncNR4A3 over-expressing K-562 and empty vector control cells shows significant modulation of NR4A3 at mRNA level in K-562 (mean ± SEM, n=3), similar results for KG1 cells are shown in **Supplementary Figure 8**. **(B)** CCK-8 viability assay results showing reduced viability after lncNR4A3 over-expression in k-562, results for KG1 are shown in **Supplementary Figure 8**. **(C)** Photographs of human hematopoietic CFUs in semi-solid culture (bar: 200µm), see additional colonies in supplementary information. Left: representative colony from control CD34+ cells after 15 day in culture. Right: representative cluster from LncNR4A3 over-expressing cells 15 days in culture. **(D)** Immunoblots showing the reactivation of NR4A3 protein in AML CD34+ cells, KG1 and K-562 cells. * : <0.05, *** : <0.001.

The endogenous expression of both *NR4A3* (data not shown) and lncNR4A3 in myeloid cell lines is almost completely abrogated (see **Supplementary Figure 4**), therefore we sought to determine if the inactivation of *NR4A3* could be reversed by lncNR4A3 artificial re-expression. Transient over-expression of lncNR4A3 led to a more than 2-fold increase of *NR4A3* mRNA (**Figure 3A** for K-562 and **Supplementary Figure 8** for KG1) and more importantly, this regulatory effect translated into an increased NR4A3 protein level (**Figure 3D**). Levels of lncNR4A3 and NR4A3 mRNA were evaluated by qRT-PCR. Efficiency of the over-expression was verified by qRT-PCR, which showed that all nucleofections rendered more than 500-fold over-expression of lncNR4A3 compared to controls (see **Supplementary Figure 7**).

Consistent with this reactivation of *NR4A3*, the proliferation of K-562 and KG1 cells was reduced as shown by cell viability assay CCK-8 (**Figure 3B** and **Supplementary Figure 8**, K-562 and KG1 respectively). We also examined the effects of lncNR4A3 in CD34+ hematopoietic cells from two acute myeloid leukemia patients. Expression of NR4A3 mRNA was increased in both samples after lncNR4A3 over-expression (38% and 68% increase in mRNA level) and due to sample availability, only one patient sample was analyzed by western blot, confirming upregulation of *NR4A3* (**Figure 3C**). Nucleofection-mediated transient over-expression of lncNR4A3 in these cells caused an important reduction in their colony formation capacity (15-days methylcellulose CFU assay) when compared to empty-vector nucleofected cells (**Figure 3D**). Methylcellulose colony-forming unit (CFU) assay shows that the over-expression of lncNR4A3 compromised the proliferation capacity of these cells, evidenced by the reduced number of colonies as well as colony size in lncNR4A3-nucleofected cells after 15 days in semi-solid culture (control-1: 7 clusters, 5 colonies, lncNR4A3-1: 5 clusters, 0 colonies; control-2: 19 clusters, 13 colonies, lncNR4A3-2: 2 clusters, 1 colony) see **Figure 3D** and **Supplementary Figures 8** and **9**.

## Discussion

Despite the advancements in therapeutic interventions, acute myeloid leukemia (AML) patients have limited treatment options and mortality remains high. Myelodysplastic syndromes (MDS) are a group of pre-leukemic conditions, associated to aging, characterized by inefficient hematopoiesis and accumulation of genetic lesions (31). There is a diversity of genetic abnormalities associated with leukemogenesis, and targeting a mechanism common to myeloid malignancy is a challenge to the development of efficient therapies. The suppression of NR4As nuclear receptors is a common feature of AML irrespective of subtype and cytogenetics, and the loss of these nuclear receptors leads to rapid development of AML in mice. All these characteristics make the reactivation of *NR4A1/3* an appealing treatment approach. However, the mechanisms that regulate *NR4A1/3* repression during myeloid malignization are not well-understood.

Heterogeneous nuclear ribonucleoproteins (HnRNP) comprise a family of multifunctional RNA binding proteins involved in different levels of transcriptional and post-transcriptional regulation including pre-mRNA processing, mRNA stability, and translation (32–36). Several members of this family have been associated with RNA elongation and splicing (32, 34, 36). Moreover, *HnRNPK* has been characterized as a tumor suppressor in myeloid malignancy (37). This protein family, as well as other proteins identified in this study as targets of lncNR4A3, are associated with RNA synthesis, elongation and splicing (25, 27, 28, 32, 34).

Here, we identified a novel sense non-coding transcript in the *NR4A3* locus. The expression of this transcript is abrogated in myeloid malignancy patients, and myeloid malignant cell lines. Finally, the artificial re-expression of lncNR4A3 caused the re-activation of *NR4A3* in leukemic cells and modulation of several proteins associated to RNA processing in K-562.

Although, we embarked on the characterization of lncNR4A3 under the hypothesis of a cis-regulatory role of lncNR4A3 upon *NR4A3*. Our results provide evidence of a broader reach of this transcript in the regulation of a set of proteins involved in RNA processing. Unfortunately, proteomics approaches are not as comprehensive as transcriptomic ones and due to sensitivity limitations of the technique and equipment, we cannot infer that these proteins are directly regulated or the only ones regulated by lncNR4A3. We also cannot conclude that the dysregulated proteins are cause or consequence of NR4A3 dysregulation. Despite the numerous reported cases of local regulation by long non-coding RNAs (12, 38–41), results from global transcription analysis reveal that long non-coding RNAs act as global expression regulators, modulating, not only one, but many distant loci (15, 38, 41–43). Moreover, there are several cases of lncRNAs acting through cis- and trans-mechanisms simultaneously (38, 40, 41). Deeper analysis of several cases of reportedly cis-acting lncRNAs revealed a wider set of genome-wide targets from which the locally regulated gene was merely a part or an indirect product of a *trans*-mechanism (38, 40, 43). All these are possible scenarios of the regulatory landscape of the *NR4A3* locus. In this study we have pointed to a completely new player in the regulation of NR4A3 and the association of this lncRNA with RNA processing machinery. However, future work is necessary to identify direct targets of this transcript and precise regulatory mechanisms of this important locus in the light of these new findings.

Another technical limitation of the study of LncNR4A3 is the fact that primer design cannot exclude immature forms of *NR4A3* as contaminants in the qRT-PCR quantification. We performed an amplification using primers spanning exon 3 and adjacent intron to quantify the influence of non-processed *NR4A3* RNA (**Supplementary Figure**). We were able to detect the immature *NR4A3* RNA (exon-intron primers); however, close to the limit of reliable detection and in significantly less abundance (~3 cycles) than lncNR4A3.

Despite these limitations, the strong functional association between the up-regulated proteins, suggests a role of lncNR4A3 in RNA processing. We reviewed the literature to understand the nature of the mechanisms that regulate *NR4A3* expression. Although some reports suggested that epigenetic modification is involved in the silencing of *NR4A3* in some cancer models (1, 44), solid evidence from a recent study demonstrated that the abrogation of *NR4A3* expression in myeloid malignization was mediated by the blockade of transcriptional processing rather than epigenetic silencing (30). They showed that *NR4A3* promoter region of AML blasts lacks common epigenetic markers of repression compared to normal cells, and their repression during malignization depends on RNA processing defects (30). Here, we present evidence of the role of this novel long non-coding RNA, lncNR4A3 in *NR4A3* regulation and the modulation of a set of RNA processing related proteins. This study focused on the effect of this new regulatory transcript in myeloid malignancy; however, *NR4A3* plays important roles in lymphopoiesis (9) and its suppression is associated to lymphomagenesis (6). The reactivation of *NR4A3* by lncNR4A3 could have similar tumor suppressor effect in the context of lymphoma and other malignancies.

Our results suggest that the re-expression of lncNR4A3 is able to revert, to some extent, the loss of *NR4A3* in leukemic cells. This enhanced synthesis of *NR4A3* leads to the expected reduction in cell viability. Although, it is likely that lncNR4A3 is a fine tuner of expression for several targets, far beyond *NR4A3*, we present evidence of a tumor suppressor role of this transcript in myeloid leukemia through the regulation of *NR4A3*.

## Data Availability Statement

Proteomics quantitative data is available in a **Supplementary Material**, and raw spectra for the proteomic analysis is available upon request. Novel LncNR4A3 sequence was deposited in the NCBI database under the accession number MK510719.

## Ethics Statement

The studies involving human participants were reviewed and approved by Campinas State University Ethics Committee (number CEP1209/2011). The patients/participants provided their written informed consent to participate in this study.

## Author Contributions

AC designed and performed the research, analyzed data and wrote the paper. FN contributed in performing the research. AD contributed in performing the research. KF contributed in performing the research. SO-S contributed in the design of the research, data analysis, and interpretation. All authors contributed to the article and approved the submitted version.

## Funding

This work was supported by the Fundação de Amparo à Pesquisa do Estado de São Paulo (FAPESP) and carry out in the Hematology and Transfusion Medicine Centre – UNICAMP. The authors would like to gratefully acknowledge Romenia Ramos Domingues, Dr. Bianca Alves Pauletti and Dr. Adriana Franco Paes Leme at the Brazilian Biosciences National Laboratory CNPEM (Campinas, Brazil) for providing technical support, and the Brazilian Biosciences National Laboratory (LNBio, CNPEM), for their support with the use of the Q-tof spectrometer for the proteomics analysis.

## Conflict of Interest

The authors declare that the research was conducted in the absence of any commercial or financial relationships that could be construed as a potential conflict of interest.
