# Supplementary material for: Novel Non-Coding Transcript in NR4A3 Locus, LncNR4A3, Regulates RNA Processing Machinery Proteins and NR4A3 Expression
Source: Front Oncol. 2020 Nov 23;10:569668. doi: 10.3389/fonc.2020.569668 (PMC7719789; doi:10.3389/fonc.2020.569668)

**Supplementary material**

Novel non-coding transcript in NR4A3 locus, LncNR4A3, regulates RNA processing machinery proteins and NR4A3 expression

A.Congrains, F.S. Niemann, A.S. Duarte, K.P. Ferro, S.T. Olalla-Saad

Hematology and Hemotherapy Center, Hemocentro­Unicamp, Campinas, São Paulo, Brazil

Corresponding author: Ada Congrains, email: [adacongrains@hotmail.com](mailto:adacongrains@hotmail.com), mailing address: Rua Carlos Chagas 480, Barao Geraldo, Campinas, SP, Brazil/Unicamp, phone number : +55(19) 32895438

**Supplementary Materials and Methods**

**Cell culture and nucleofection**

K562 cells were maintained in RPMI 1640 medium supplemented with 10% fetal bovine serum. Bone marrow mononuclear cells from the acute promyelocytic leukemia patient (AML-M3) had more than 80% CD34+ cells and were directly used for the experiments described here. Cd34+ cells from the bone marrow mononuclear cells of the acute myelomonocytic leukemia patient (AML-M4) were separated using Indirect CD34 MicroBead kit, Miltenyi Biotech GmbH, Germany. Cells (1x106 per nucleofection) were resuspended in P3 Primary Cell 4D-Nucleofector™ X Kit buffer and nucleofected using Amaxa™ 4D-Nucleofector™. After nucleofection cells were maintained in Stem spam medium supplemented with 10ng/ml of thrombopoietin (TPO), stem cell factor (SCF) and FlT3-ligand (FL), interleukin 3 (IL-3) and IL-6 in culture until RNA collection (48h) or plated in Methocult® H4534 Classic without EPO for 15 days for Colony formation Unit (CFU) assay (2x10^4^ cells in 1 ml of methylcellulose medium in a 6-well plate).

**Supplementary table 1. qRT-PCR primers list**

| Gene name | Primer forward | Primer reverse |
| --- | --- | --- |
| LncNR4A3 | AAGGCCCTGCTTGTTAAAGAAA | TCTGATTGAGTCATGCATGTATGG |
| NR4A3 | AAGAGACGTCGAAACCGATG | GGTTCCTGTTGTAATGGGCT |
| HPRT (endogenous control) | GAACGTCTTGCTCGAGATGTGA | TCCAGCAGGTCAGCAAAGAAT |
|  |  |  |

**Poly A enrichment/depletion**

To separate poly A transcripts from non-polyadenylated transcripts, we used Absolutely mRNA oligo (dT) Magnetic Particles (Agilent). 4 ug of RNA were diluted to 25 ul, denatured at 65 °C and after incubation with the dT beads, supernatant (non-bound RNA) was collected as poly A depleted RNA. Quantification of this fraction yielded around 3.8 ug of RNA. Next steps were carried out according manufacturer´s instructions and poly A fraction yielded 190 ng of RNA. Both fractions were diluted to the same volume and same amounts were used for reverse transcription. RT-PCR was performed using primers showed above.

**Rapid amplification of cDNA ends**

5′/3′ RACE Kit, 2nd Generation, Roche was used to identify the 3’ and 5’ ends of lncNR4A3 from HS5 total RNA, using dT oligos for the reverse transcription as described by the manufacturer.

RACE primers

| Primer name | Sequence | Information |
| --- | --- | --- |
| 5’RACE primer SP1 | TTACATGTGCACACTGGC | For reverse transcription |
| 5’RACE primer SP2 | TGTGCACACTGGCTATAAAA | First PCR (5’) |
| 5’RACE primer SP3 | TATTGGTGTATTTGTATTTCTCTTAGG | Nested PCR (5’) |
| 3’RACE primer SP5 | CAACTGGACAGCAAGATAAGTA | PCR 3’ |

**Supplementary Results**

**LncNR4A3 characterization**

**Supplementary figure 1.** UCSC genome browser view of LncNR4A3 region. Blast of LncNR4A3 complete sequence. Red box: ESTs identified in this region.


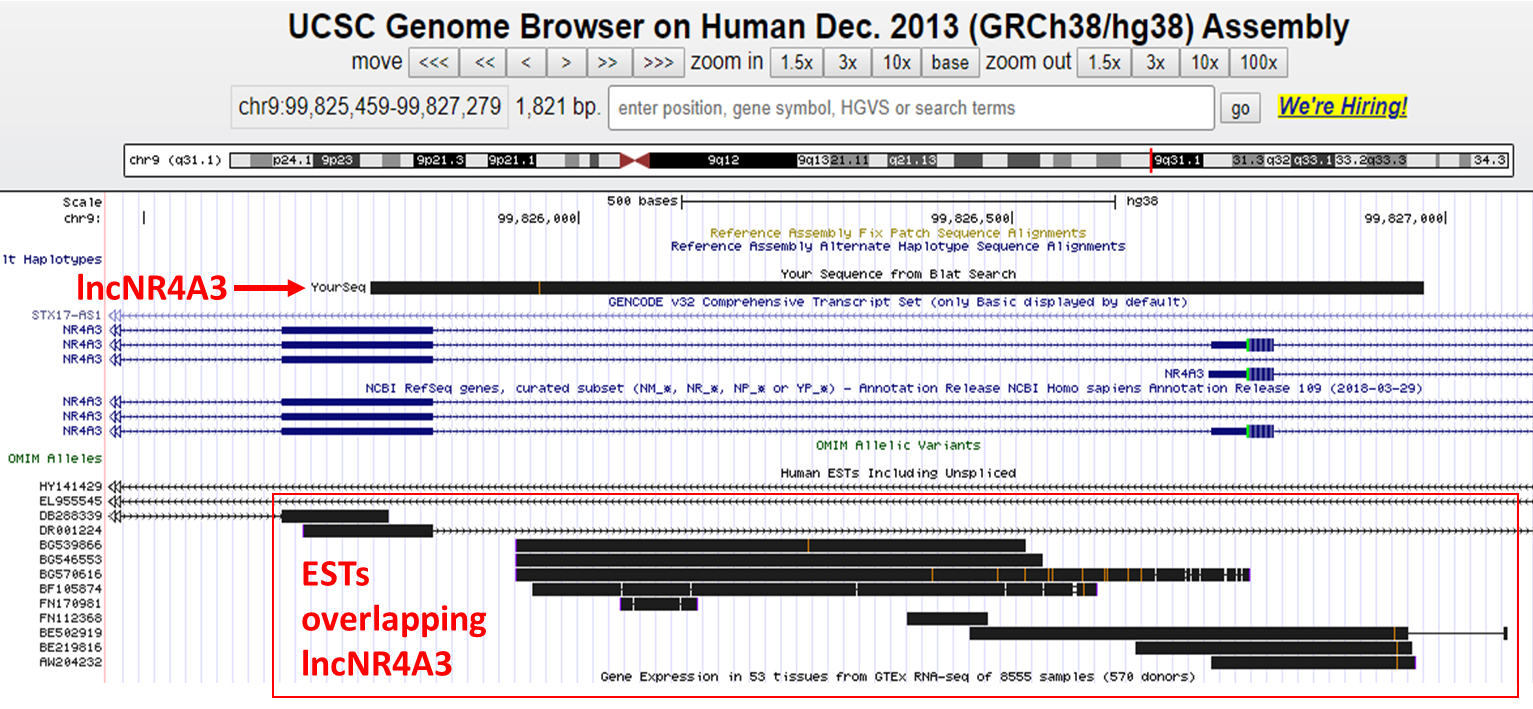


After Rapid amplification of cDNA ends (RACE), complete sequence obtained from cDNA of HS5 cell line:

Position of cloning primers underlined in the sequence 🡪

LncNR4A3 cloning primer forward: CTGGCCGCGAATTTCACAAC

LncNR4A3 cloning primer reverse: AAACGGCGGTTTTTGGTGG

**FASTA – 5’ – 3’ chr9: 99825762- 99826714 strand + 1214bp**

TCCTCCTACACTCTCAGCCTCCGCTGGAGAGACCCCCAGCCCCACCATTCAGCGCGCAAGATACCCTCCAGGTAGGTCCGAAGGCAAGACCCTTTTCTCCTCCCTGGCTGAGGGAAGTGGGTGGGGGAACCACACACTCGGCGGGCAGCGTGGTCGACCTGCCCAGTGCCAGGACAGTGACTGCTGGCCGCGAAATTTCACAACACAGGTGGCTTCCTCACAGGAAGCTCCTCTGTATACCACACCCTGTTGCTACTGAGTGGAGCAGCCAAATTAAATTAAGCTTGCATTGCTCAAAATTAATTTTCCTAAGAGAAATACAAATACACCAATAGATTAGGGTATTTTATACATTTTTAATTTCATTTTTGCTCTTCTTTTATAGCCAGTGTGCACATGTAAGAGTTATGGAATCACTTAGAGCAATACTGAGCATTTTCATTTATATAAAACCCAAATCATTTGGGTGCAGAAGTTTGGATGATTGAAGCTCAGAGGGAAGGAGAAAAGCATTTGAGATGAAAACCAAAGAGTAAATTTGAGTTTGGCAAAAGAACAGATTGCACTTTTGGTTTGTACCACCTCTTTACAAATTTGTTAAAGGAATACTAGGTGCCAAGCCTGTGTGGGCACCATAGATAATGCAGATAAATAGAATACAATCCCAGCAAGCTCATCTTCTAGCTAGAAACCTCAGAAAGACACAAATAAGAGCAGTAAAAAGGGATTGGCCGGAAAGAATGACAGAAAAGAGTAATGGAGAAATCCTAGAGGTGCTGAAGGGAGAAGGAGAATAGATCAAAGATCAGGCAGTGTTCTTTCTTTTAAATGATTAGCCTTTCATTTCATCCCAACAACTGGACAGCAAGATAAGTAACTGGACTTCAAACTAGTGAGTGTATTTTTAAGGCCCTGCTTGTTAAAGAAAGGCTTGAACTGGCCTCTCCTCATCACTGCTTCTTCCAACAGGCCCTCATCACCTTTTTTCAAGTCAAGATTTCATCCCATACATGCATGACTCAATCAGATTTGGAAATGTGGGTAAGAGAAAGATGTCAAAGGAAATGTGAAGTATTCACTTCTCTATTAGTCACACCTTTTACACCATAGACTCCAAAGAGGCGTTAAGCACCTGGTTTTCCTTTGGCTCAGAAAAACCAACCACCAAAAACCGCCGTTTTTTACCATTTATATTTAGCCATAAAGAAAGAAAAA

**LncNR4A3 is a polyadenylated transcript (it amplifies from poly A enriched RNA):**

LncNR4A3 only amplified in the poly A enriched fraction for cDNA from K562, while coding NR4A3 only amplified in the poly A enriched fraction.

**Supplementary figure 2.** Amplification plot and agarose gel showing the PCR product band of the RT-PCR using the poly A- enriched fraction and Poly A -depleted fraction of RNA.


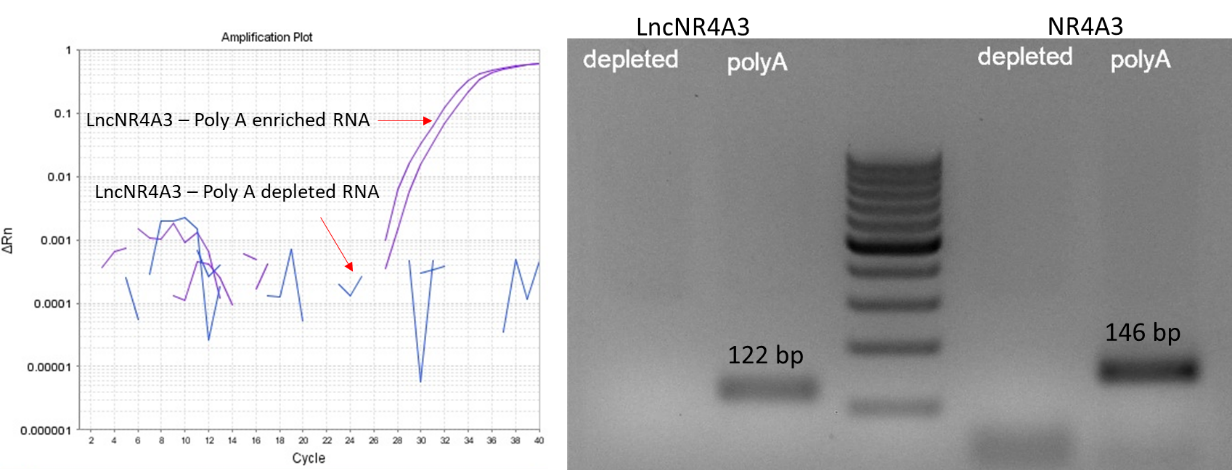


**qRT-PCR detection of immature forms of the NR4A3 gene**

Transcription and RNA processing are occurring simultaneously in the cell and consequently we expect unprocessed forms of coding genes to be very transient and very low in expression. However, due to the impossibility to design primers for LncNR4A3 (intronic sense transcript) that would exclude immature forms of NR4A3, we carried out an RT-PCR to quantify the immature form of NR4A3 using primers spanning an exon -intron region (Forward TGCAAGGGCTTTTTCAAGGT Reverse ACAGCTCTCAAACCGGGAAGA). We observed at least 3 cycles difference between the amplification of the unspliced NR4A3 and lncNR4A3 using the same k562 cDNA sample.

**Supplementary figure 3.** Amplification plot using LncNR4A3 primers (details showed in lower panel) and NR4A3 Exon-Intron (spanning Exon 3 and adjacent intron) primers.

**
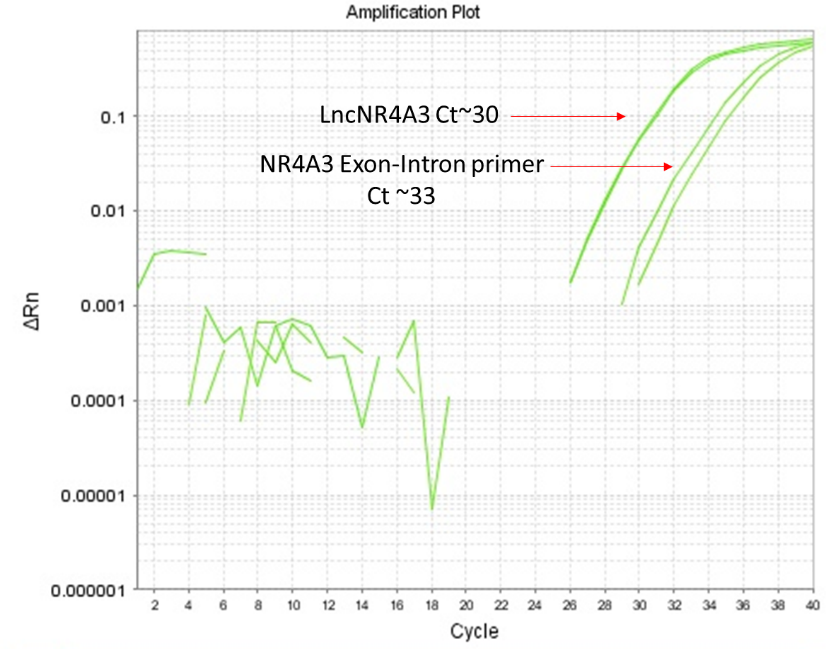
**


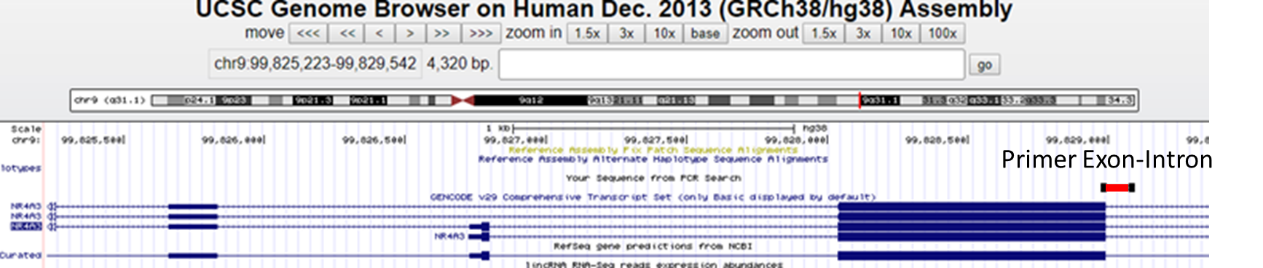


**Supplementary figure 4. Endogenous expression of lncNR4A3 in malignant cell lines and normal CD34+ Hematopoietic stem cells**

Q-RT-PCR was used to quantify expression of lncNR4A3 in several myeloid malignant cell lines (p39, k562, ku812, u937), lymphoid cell lines (Daudi, Raji) and normal CD34+ hematopoietic stem cells from umbilical cord. Amplification of lncNR4A3 in myeloid cell lines was close to the limit of detection of the technique (over 30 cycles).


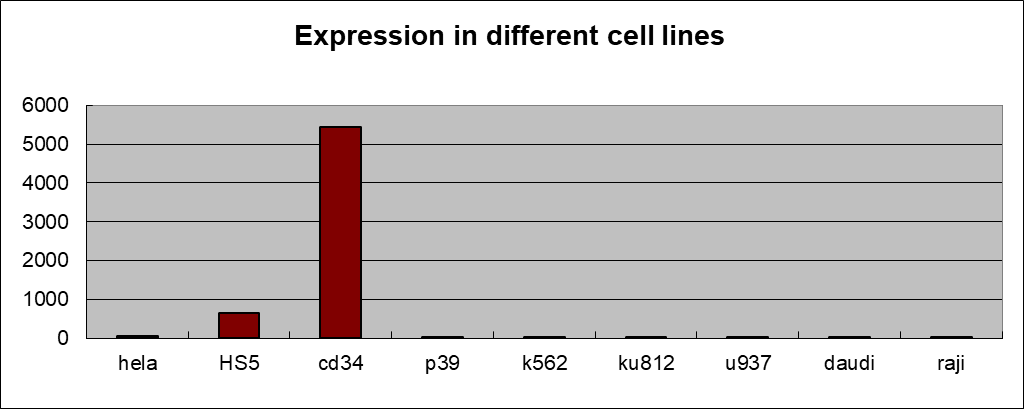


**Nucleofection efficiency**

**Supplementary figure 5.** Nucleofection efficiency of K562 cells was evaluated by running a nucleofection using pmax-GFP provided by the nucleofection kit manufacturer. 48h after the nucleofection cells were washed twice with PBS and flow cytometric analysis was performed using FACSCalibur and analysed using Modfit software. Viable cells were gated by morphology using Modfit sofware and nucleofection efficiency (GFP positive cells) was estimated in 40% of viable cells.


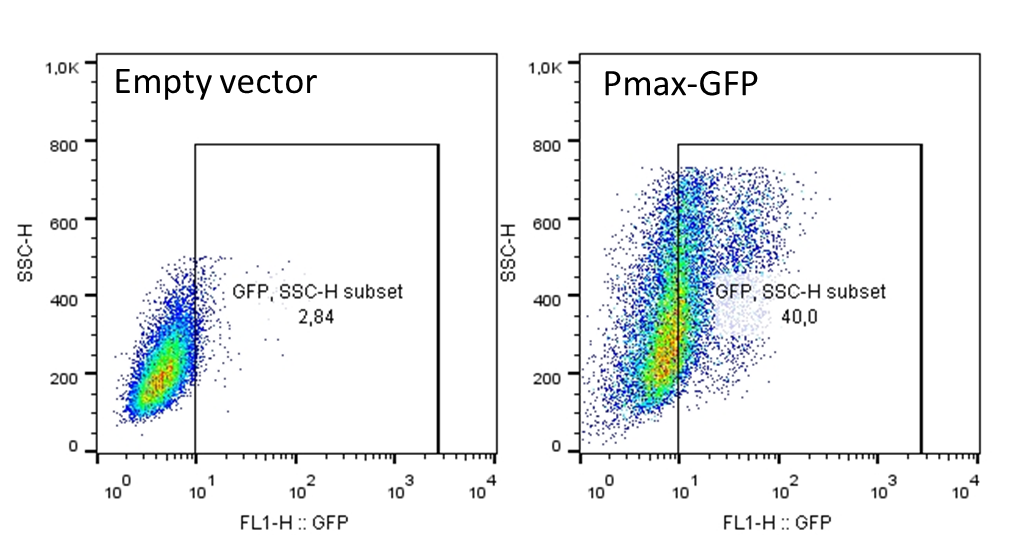


**Supplementary figure 6.** Positive control for the nucleofection of CD34+ cells was carried out as described above. Viable cells were gated by propidium iodide (PI) negative staining and GFP positive cells was estimated in around 40% of viable cells.


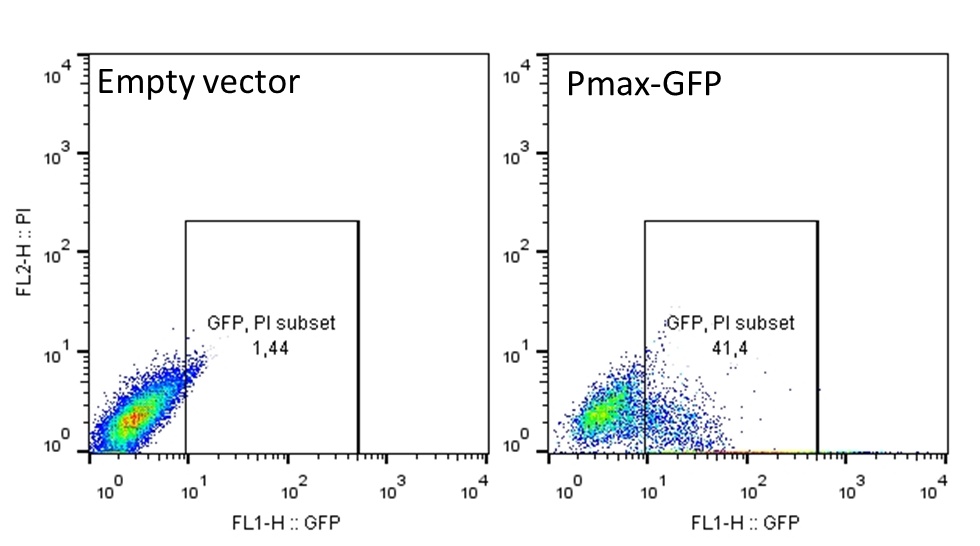


**Over-expression efficiency by qRT-PCR**

LncNR4A3 over-expression was verified by qRT-PCR. All nucleofections rendered more than 500-fold over-expression of lncNR4A3, as shown below.

**Supplementary figure 7.** Relative quantification of lncNR4A3 expression in k562, KG1 and CD34+ cells from AML patients.

**Over expression of lncNR4A3 in KG1**

**Supplementary figure 8.** Over-expression of lncNR4A3 in KG1 cell line led to the reactivation of NR4A3 in mRNA and concomitant reduction in cell growth (n=2, P<0.01 and P<0.05 respectively).


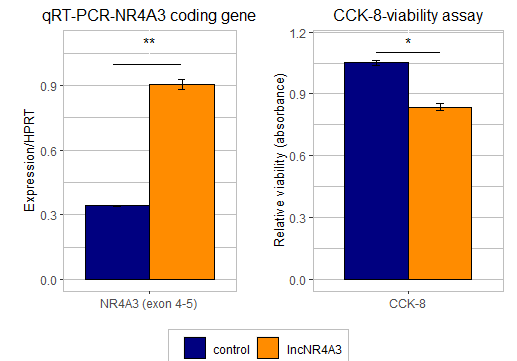


**HnRNPK protein level after lncNR4A3 over-expression**

**Supplementary figure 9.** Due to the previously known association between HnRNPK and NR4A3 expression, we evaluated protein level of HnRNPK in all cells analized. HnRNPK was up-regulated in K-562, as shown in figure 2 and supplementary figures, also in AML CD34+ cells, but we could not detect this up-regulation in KG1 as shown below.


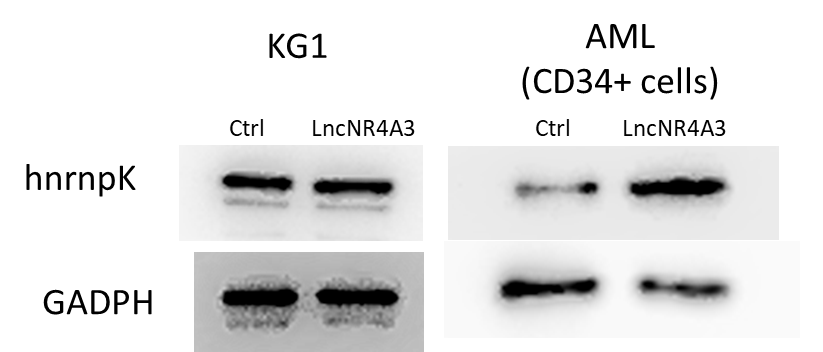


**Additional microscopic photographs**

**human hematopoietic CFUs**

**Supplementary Figure 10.** Representative CFUs formed by control- empty vector over-expressing cells after 15 days in semi-solid culture (bar : 200µm) :


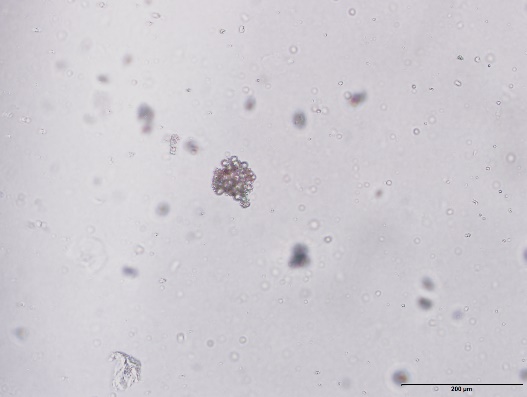

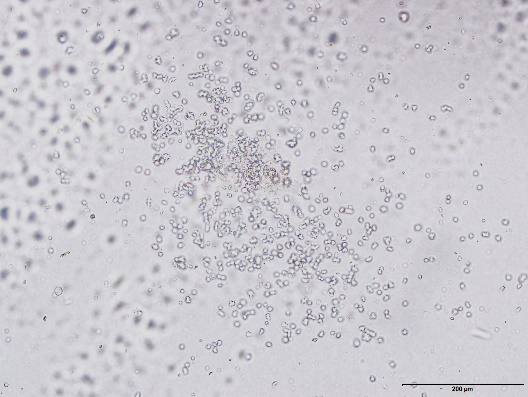

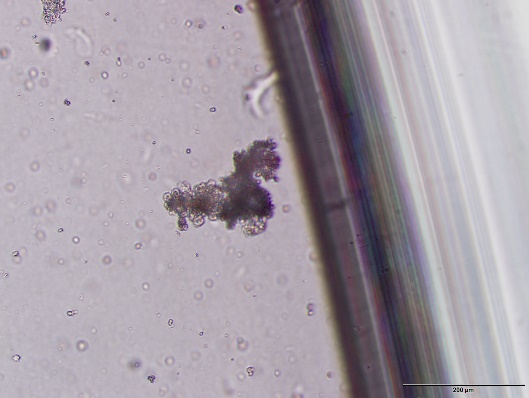


**Supplementary Figure 11.** Representative CFUs formed by LncNR4A3 over-expressing cells after 15 days in semi-solid culture (bar : 200µm):


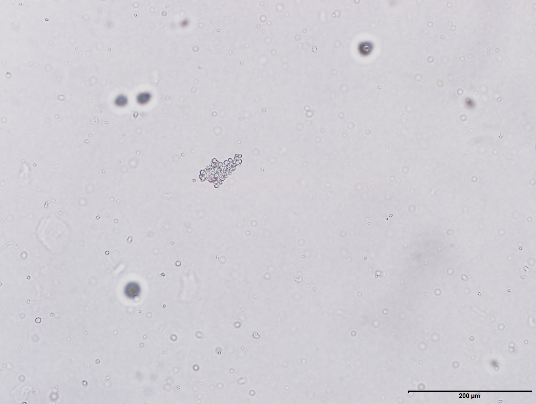

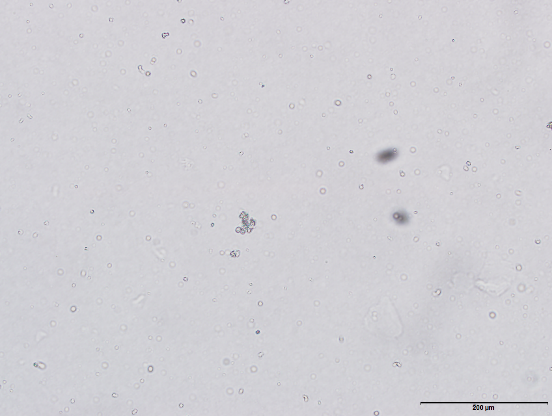

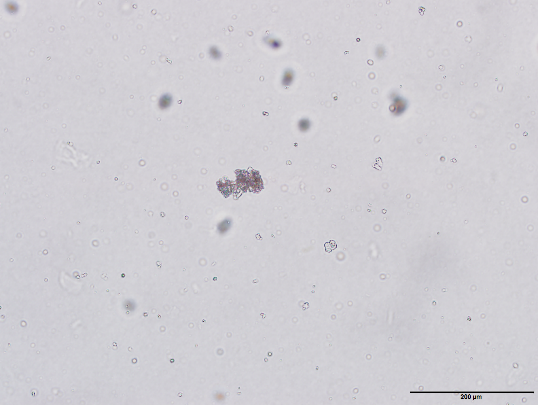


**Western blots**

Blots of 3 independent experiments of nucleofection of K562. We detected up-regulation of NR4A3, hnRNPK, hnRNPA1, SF3B2, Lamin B1 and PARP1, and downregulation of hemoglobin subunit alpha, which signal was difficult to quantify and was excluded from the main manuscript, they are consistent with the MS-proteomics results.

**Supplementary Figure 12.** Immunoblots for 3 independent experiments using cell lysates of controls and LncNR4A3 over-expressing K562 as described in methods.

Gel 1


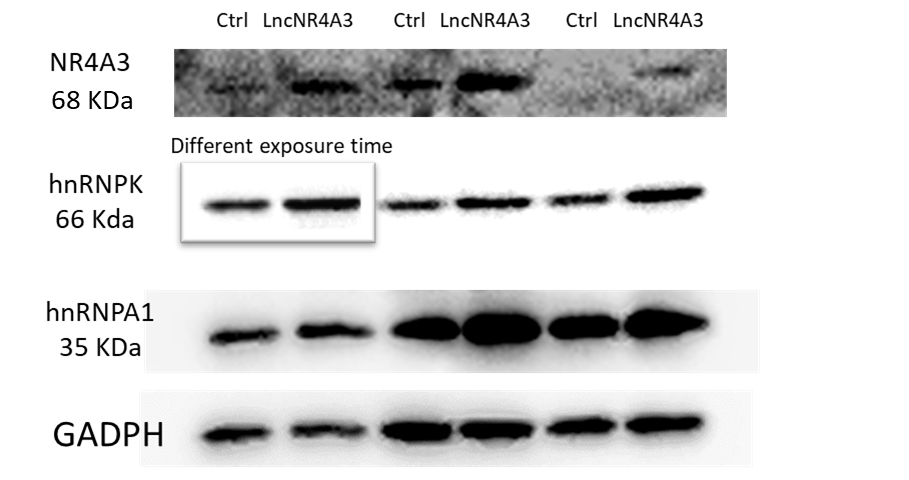


Gel 2


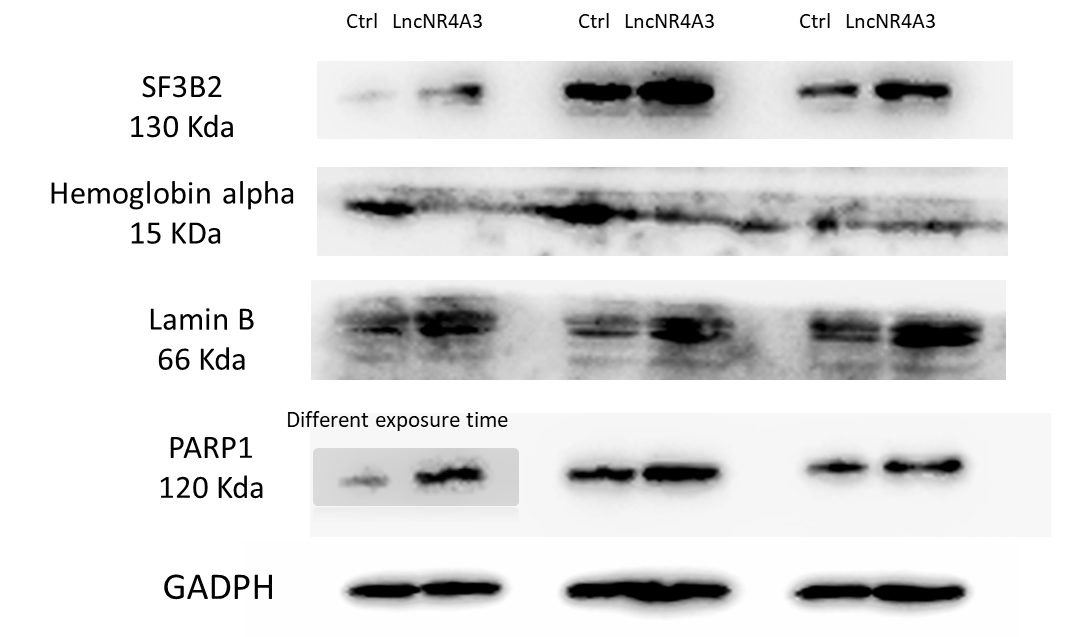

Supplement: Supplementary file 1 [file DataSheet_1.docx]
